# Transcriptomic Assessment of PD-L1 (*CD274*) in NSCLC: Biological Significance, Clinical Relevance, and Methodological Challenges

**DOI:** 10.3390/biomedicines14102318

**Published:** 2026-10-12

**Authors:** Maciej Baron, Iwona Jabłońska, Piotr Lewandowski, Małgorzata Wachowicz, Eliza Tomkiewicz, Adam Skrzypiec, Bogna Drozdzowska

**Affiliations:** 1 Department of Pathomorphology, Faculty of Medical Sciences in Zabrze, Medical University of Silesia in Katowice, 40-055 Katowice, Poland; bdrozdzowska@sum.edu.pl; 2 IIIrd Radiotherapy and Chemotherapy Department, Maria Skłodowska-Curie National Research Institute of Oncology, 44-102 Gliwice, Poland; iwona.jablonska@gliwice.nio.gov.pl; 3 Department of Histology and Cell Pathology, Faculty of Medical Sciences in Zabrze, Medical University of Silesia in Katowice, 40-055 Katowice, Poland; d201142@365.sum.edu.pl (P.L.); s86293@365.sum.edu.pl (M.W.); s86275@365.sum.edu.pl (E.T.); aaskrzyp@gmail.com (A.S.)

**Keywords:** non-small cell lung carcinoma (NSCLC), PD-L1, *CD274*, PD-1, *PDCD1*, transcriptomics, RNA expression, immunohistochemistry, prognosis, biomarkers

## Abstract

PD-L1 immunohistochemistry is widely used to guide immune checkpoint inhibitor therapy in non-small cell lung carcinoma (NSCLC), but its clinical performance is limited by spatial and temporal heterogeneity and assay-related variability. Transcriptomic assessment of *CD274* may provide complementary information. We conducted a narrative review of PubMed to evaluate the biological and clinical significance of *CD274* transcript expression in NSCLC, with additional consideration of *PDCD1*/PD-1 and methodological sources of heterogeneity. Overall, 29 studies were included, predominantly retrospective cohorts. *CD274* transcript levels generally showed a positive, but variable, association with PD-L1 protein expression. Associations with histological subtype, tumour stage, smoking status, molecular alterations, and survival were inconsistent across studies. Treatment-associated findings were more promising in selected immune checkpoint inhibitor-treated cohorts. Associations were more pronounced in selected co-expression-defined subgroups, particularly for *CD8A/CD274*; however, the incremental value of multimarker models over single-gene assessment has not been consistently established. Differences in cellular composition, spatial heterogeneity, treatment exposure, reference-gene selection, and tissue preservation likely contributed to discordant results. In conclusion, *CD274* transcript expression may complement PD-L1 immunohistochemistry in NSCLC, but current evidence is insufficient to support its use as a stand-alone prognostic or predictive biomarker. Prospective validation and methodological standardisation are required.

## 1. Introduction

Lung cancer remains the leading cause of cancer-related mortality worldwide, accounting for approximately 1.8 million deaths annually [1]. Non-small cell lung carcinoma (NSCLC) represents approximately 85% of all lung cancer cases [1,2]. Rather than constituting a single disease entity, NSCLC comprises a heterogeneous group of histological subtypes, predominantly adenocarcinoma (AD) and squamous cell carcinoma (SCC), as well as large-cell carcinoma and other less common entities. These subtypes differ in their molecular characteristics, biological behaviour, and clinical outcomes [3]. 

**Figure 1:**
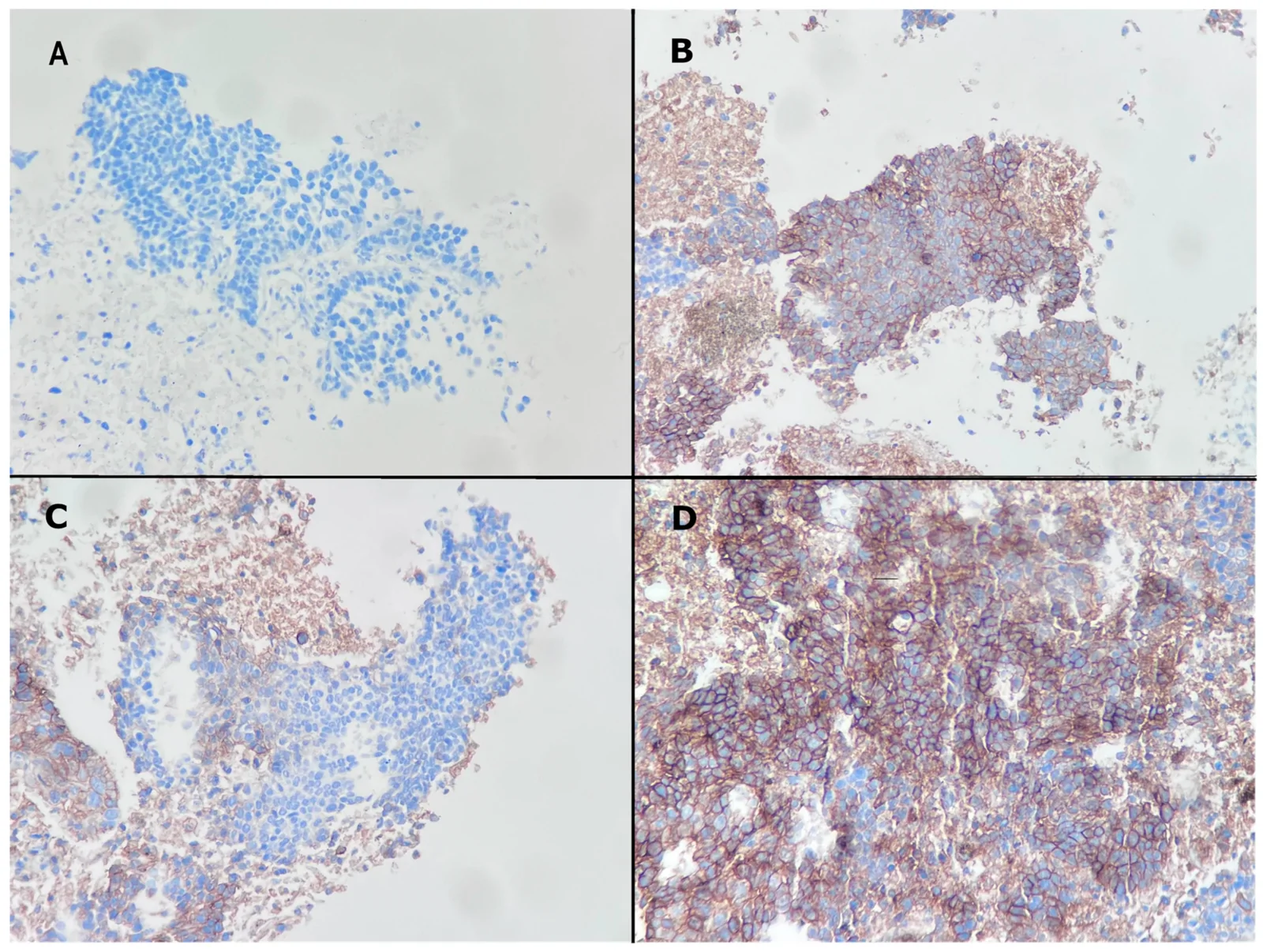
Representative PD-L1 immunohistochemical staining in archival FFPE lung adenocarcinoma specimens. The figure illustrates the diagnostic Tumour Proportion Score (TPS) categories: <1%, 1–49%, and ≥50%. Low expression (TPS <1%) (**A**), intermediate expression (TPS 1–49%) (**C**), and high expression (TPS ≥50%) (**B**,**D**). Original magnification ×100 (**A**–**C**) and ×200 (**D**). The slides were prepared as part of routine diagnostic immunohistochemistry performed at the Department of Pathomorphology, Medical University of Silesia in Katowice, and were subsequently retrieved from the departmental archive for illustrative purposes. PD-L1 staining was performed using the 22C3 antibody on the Dako Autostainer Link 48 (Agilent Technologies) platform, and TPS categories were confirmed by a pathologist. Scale bars could not be reliably reconstructed because the archived micrographs did not retain calibrated spatial metadata.

The PD-1/PD-L1 axis is a major immune checkpoint pathway involved in maintaining peripheral immune tolerance. PD-1 and PD-L1 are encoded by *PDCD1* and *CD274*, respectively. PD-1 is expressed on activated immune cells, including T cells, B cells, natural killer cells, and monocytes. PD-L1 is broadly expressed on both haematopoietic and non-haematopoietic cells, including B cells, dendritic cells, macrophages, vascular endothelial cells, and epithelial cells. Engagement of PD-1 by PD-L1 inhibits intracellular signalling pathways, including the phosphatidylinositol 3-kinase (PI3K)/Akt pathway, thereby suppressing immune-cell activation, proliferation, and survival. Consequently, the PD-1/PD-L1 axis represents an important mechanism of immune evasion in NSCLC [4]. Accordingly, elevated PD-L1 protein expression is frequently observed in NSCLC tissues [5,6]. Representative immunohistochemical staining for PD-L1 in lung adenocarcinoma is presented in Figure 1.

Despite considerable advances in diagnosis and treatment, the prognosis of NSCLC remains strongly dependent on tumour stage and continues to be particularly poor in patients with advanced disease [2]. The therapeutic landscape of NSCLC has been transformed by the introduction of molecularly targeted therapies and immune checkpoint inhibitors (ICIs). Molecularly targeted therapies are effective primarily in patients whose tumours harbour actionable genomic alterations, whereas ICIs targeting the PD-1/PD-L1 axis aim to restore antitumour immune responses. Nevertheless, the efficacy of both treatment strategies is limited by primary and acquired resistance [7]. In the case of PD-(L)1 blockade, more than 60% of patients who initially respond to treatment may eventually develop acquired resistance [8]. Consequently, there is an ongoing need for biomarkers that can improve patient stratification and provide clinically relevant prognostic or predictive information.

PD-L1 protein expression assessed by immunohistochemistry remains the most widely implemented biomarker for guiding the use of PD-1/PD-L1 inhibitors in NSCLC [9]. However, its clinical performance is limited by spatial and temporal heterogeneity, assay-dependent variability, and the occurrence of responses in some patients with PD-L1-negative tumours [10,11,12]. Importantly, mRNA abundance does not necessarily correspond to the abundance of the encoded protein, indicating that transcriptomic and protein-level measurements may capture partly distinct biological information [13]. Moreover, the clinical significance of PD-L1 assessment has predominantly been investigated at the protein level, whereas evidence concerning its transcriptomic expression and prognostic relevance remains fragmented. 

Recent reviews have addressed several related aspects of PD-L1 assessment in NSCLC. Qi et al. provided a broad overview of available PD-L1 detection approaches, including immunohistochemistry, liquid biopsy, genetic assays, molecular imaging, and artificial intelligence-based methods [14]. Ferrari et al. focused primarily on the molecular regulation of PD-L1 expression and emerging multimodal biomarker strategies [15], whereas Soo et al. systematically evaluated the clinical utility of different PD-L1 protein assays in patients receiving immune checkpoint inhibitors [16]. Although these studies provide important complementary perspectives, they do not primarily address tissue *CD274* transcript expression as a distinct analytical and clinical biomarker or integrate RNA–protein concordance, clinical associations, and methodological sources of RNA-based heterogeneity. The present review therefore focuses specifically on these unresolved aspects of *CD274* transcript assessment in NSCLC.

This narrative review therefore addresses three principal questions: (1) To what extent does tissue *CD274* transcript expression correspond to PD-L1 protein expression assessed by immunohistochemistry? (2) What evidence supports associations of *CD274* and *PDCD1* transcript expression with clinicopathological characteristics, prognosis, and treatment-associated outcomes? (3) Which biological and methodological factors may account for discordance between transcript- and protein-based assessment and between published studies? Particular attention is given to the potential clinical role and current limitations of RNA-based PD-L1 assessment in NSCLC. 

## 2. Search Strategy

This narrative review evaluated the available evidence on the biological and clinical significance of *CD274* transcript expression in NSCLC, while also considering *PDCD1*/PD-1 and the potential biological and methodological sources of discrepancies among published studies. 

PubMed was initially searched up to 26 July 2026, with no restriction on publication date, and the search was updated on 16 September 2026. The updated search identified two additional eligible studies. No restriction to specific PubMed search fields was applied. An exploratory and iterative search approach was used to identify studies addressing *CD274* and *PDCD1* transcript expression in NSCLC and methodological factors relevant to its interpretation. Search terms included combinations of “non-small cell lung cancer”, “NSCLC”, “CD274”, “PD-L1”, “PDCD1”, “PD-1”, “mRNA”, “RNA”, “transcript”, “transcriptomic”, “RT-qPCR”, and “RNA sequencing”. Because the search was exploratory and iterative, no single fixed Boolean search string was applied; the principal terms listed above were combined flexibly according to the specific aspect being investigated. Additional targeted searches were performed for methodological factors relevant to the interpretation of *CD274* transcript expression, including RNA–protein concordance, cellular composition, spatial heterogeneity, tissue preservation, and reference-gene selection. Reference lists of relevant original studies and reviews were also examined to identify additional publications.

Original English-language studies were eligible if they assessed *CD274* and/or *PDCD1* expression at the RNA level in human NSCLC tissues or investigated methodological factors directly relevant to the interpretation of transcriptomic findings. Secondary analyses of publicly available transcriptomic datasets were included as complementary evidence. Protein-only studies were considered only when they addressed factors relevant to RNA-based assessment, such as spatial heterogeneity, cellular localisation, or tissue sampling. 

The primary focus of this review was tissue-based assessment of *CD274* transcript expression in human NSCLC, with *PDCD1* considered as complementary evidence where relevant. Transcript-level studies, paired RNA–protein analyses, secondary transcriptomic datasets, and protein-only or methodological studies were interpreted according to their directness, with protein-only evidence used solely as biological or analytical context.

Literature searching, initial screening, and data extraction were performed by M.B. and E.T. The eligibility and interpretation of the studies included in the final synthesis were subsequently reviewed by P.L. and M.W. Because this study was designed as a narrative rather than systematic review, no formal systematic-review protocol, PRISMA-based screening procedure, meta-analysis, or formal risk-of-bias assessment was undertaken.

Following the updated literature search, the narrative synthesis comprised 29 studies addressing the clinical, biological, or methodological aspects of *CD274/PDCD1* transcript assessment relevant to the scope of this review. Most available evidence originated from retrospective surgical cohorts, with relatively few prospective studies and additional evidence derived from public transcriptomic datasets and treatment-specific cohorts. A structured inventory of studies contributing to the narrative synthesis, including study design, cohort characteristics, analytical methods, principal findings, and key limitations, is provided in Supplementary Table S1.

## 3. Expression Patterns and RNA–Protein Concordance

In a retrospective cohort, *CD274* expression was notably higher in NSCLC tumour tissue than in corresponding non-neoplastic tissue [17]. In contrast, Cheng et al. reported lower *CD274* expression in the tumour than in adjacent non-neoplastic tissue, which also showed greater inferred immune-cell infiltration [18]. These contrasting observations may reflect differences in tissue composition and sampling, although the available data do not allow a definitive explanation. Moreover, *CD274* transcript levels have been associated with the composition of the tumour immune microenvironment, particularly with the abundance of macrophages, natural killer cells, and cytotoxic T cells [19].

Across available cohorts, most of which were retrospective, PD-L1 expression at the RNA and protein levels generally showed a positive relationship, although the strength of this association varied considerably between studies [13,17,19,20,21,22,23,24,25,26,27]. 

Evidence from prospective cohorts was limited and heterogeneous. In a small prospective study including 22 NSCLC cases, Erber et al. reported excellent agreement between *CD274* mRNA and PD-L1 protein expression assessed using the 28-8 antibody in primary NSCLC tissues (*n* = 8; κ = 0.85) and endobronchial ultrasound-guided transbronchial needle aspiration (EBUS-TBNA) samples (*n* = 9; κ = 1.0), whereas agreement was poor in metastatic lesions (*n* = 5; κ = 0.20), indicating substantial tissue-site dependence [25]. In a larger prospective cohort of 141 primary untreated lung cancer specimens, predominantly NSCLC (126 samples), Kumar et al. reported a significant positive association between *CD274* mRNA and PD-L1 protein expression, however, a separate NSCLC-specific correlation analysis was not reported [27]. 

In a retrospective study of formalin-fixed, paraffin-embedded (FFPE) NSCLC specimens, *CD274* expression also differed between phenotypes defined by PD-L1 expression in tumour and immune cells. Tumours with a low tumour proportion score (TPS) and high immune-cell PD-L1 expression showed significantly lower *CD274* transcript levels than tumours with high TPS, indicating that the relationship between bulk RNA expression and PD-L1 protein levels may depend on the cellular compartment assessed [23]. Similarly, Simonsen et al. found significantly lower mean *CD274* mRNA abundance in tumours classified as PD-L1-low (TPS < 50%) than in PD-L1-high tumours (TPS ≥50%) by immunohistochemistry (IHC) [28].

Importantly, the protein comparator also differed across studies. For example, Liebler et al. reported a substantial correlation between *CD274* mRNA and PD-L1 protein abundance measured by mass spectrometry, whereas agreement between mass-spectrometry-based PD-L1 measurements and IHC was weak [13].

Across the reviewed cohorts, which were predominantly retrospective, *CD274* mRNA and PD-L1 protein expression generally showed a positive association, although its magnitude varied considerably. Therefore, RNA-based assays and immunohistochemistry should not be considered fully interchangeable methods for assessing PD-L1 expression [13,17,19,20,21,22,23,25,26,27].

## 4. Association with Clinicopathological Features 

Evidence regarding *CD274* and *PDCD1* expression across the major histological subtypes of NSCLC remains heterogeneous. Among retrospective clinical cohorts, Kim et al., in a study of 687 NSCLC cases, reported higher *CD274* expression in squamous cell carcinoma than in adenocarcinoma [20], while Ludovini et al., in 191 surgically resected NSCLC cases, observed higher *PDCD1* and *CD274* transcript levels in squamous cell carcinoma than in adenocarcinoma [29]. A Cancer Genome Atlas (TCGA)-based analysis of 985 NSCLC cases similarly reported higher *CD274* expression in squamous cell carcinoma [30]. In contrast, smaller cohorts did not identify a significant association between *CD274* transcript levels and histological subtype [17,31]. Prospective evidence is limited; in a cohort of primary untreated lung cancer specimens, Kumar et al. found no significant relationship between *CD274* mRNA expression and histological subtype [27]. These studies do not establish a consistent subtype-specific expression pattern, although differences between adenocarcinoma and squamous cell carcinoma remain plausible and should be considered in future analyses [17,20,27,29,30,31].

Associations between *PDCD1* or *CD274* expression and tumour stage are similarly inconsistent. In retrospective cohorts, one study demonstrated higher transcript levels of both genes in patients with more advanced TNM stages [29], whereas another found no significant relationship between *CD274* expression and tumour stage [17]. The absence of such an association was also observed in a prospective cohort [27]. Secondary analyses of the Cancer Genome Atlas (TCGA) data have likewise produced conflicting results. One study found an association between *CD274* expression and pathological stage in lung squamous cell carcinoma but not in lung adenocarcinoma [30], whereas another TCGA-based analysis did not confirm an association in either histological subtype [32]. Because these analyses were derived from the same public data resource and may include overlapping patient populations, they should not be interpreted as fully independent replications. Overall, differences in histological composition, study design, analytical approach, and cohort overlap may contribute to the observed discrepancies.

Few studies have examined the association between smoking status and *CD274* expression. A TCGA-based analysis demonstrated an association between *CD274* expression and smoking status in lung adenocarcinoma [30], whereas a prospective cohort study found no significant association between *CD274* transcript levels and smoking history [27]. The relationship between smoking exposure and *CD274* expression has therefore not been consistently demonstrated.

*CD274* expression may also be associated with clinically relevant molecular alterations, particularly *EGFR* mutations. In a large retrospective cohort of 687 NSCLC cases, both PD-L1 protein and *CD274* mRNA expression were lower in *EGFR*-mutated tumours than in *EGFR*-wild-type tumours [20]. Complementary protein-level evidence from another large surgical cohort associated *EGFR* mutation with absence of PD-L1 protein expression; however, transcript analysis was available only in a substantially smaller subset of that cohort [21]. In a small cohort of 33 patients with *EGFR*-mutated lung adenocarcinoma, intratumoral *CD274* mRNA expression was positively correlated with *BIM* expression and inversely correlated with *VEGFA* expression [33]. However, the available evidence remains limited, predominantly retrospective, and methodologically heterogeneous; therefore, the relationship between *EGFR* status and *CD274* expression should be interpreted cautiously.

## 5. Clinical Significance

Accurate estimation of prognosis and treatment response remains an important objective in contemporary oncology. PD-L1 protein expression assessed by immunohistochemistry is widely used to support treatment selection for PD-1/PD-L1-targeted immunotherapy in NSCLC [9]. However, its role as a general prognostic biomarker remains uncertain, and the clinical significance of *PDCD1* and *CD274* transcript expression is even less clearly established.

### 5.1. Prognostic Significance

Retrospective studies have reported divergent prognostic associations for *PDCD1* and *CD274* transcript expression. Several studies reported favourable prognostic associations involving checkpoint transcript expression or transcript-derived profiles [24,34]. In surgically treated NSCLC, Usó et al. identified a favourable immunoregulatory gene-expression cluster and, separately, derived an immune checkpoint score (ICS) from weighted *PDCD1* and *CTLA4* expression. A high ICS, defined relative to the cohort median, was independently associated with longer overall survival (OS) and relapse-free survival (RFS) [34]. Similarly, Velcheti et al. reported that higher *PD-L1* mRNA expression was significantly associated with longer OS in univariable analyses in two independent NSCLC cohorts; after multivariable adjustment, the association remained significant in the Yale cohort but not in the Greek cohort [24]. Subtype-specific findings were reported by Öjlert et al.; within SCC expression subtypes, increasing *CD274* expression was associated with better prognosis in the secretory subtype (*n* = 48), whereas the association was reversed in the classical subtype (*n* = 85). No significant association was observed in the overall SCC cohort. These subtype-specific differences may partly reflect variation in the immune microenvironment, as the secretory subtype showed greater immune-cell enrichment [19].

Conversely, other retrospective studies associated increased checkpoint-gene expression with adverse clinical outcomes [29,32]. In a retrospective cohort of 191 radically resected NSCLC cases, high co-expression of *CD274* and *IDO2* was associated with shorter OS in Cox regression [29]. In another retrospective cohort of 71 surgically resected NSCLC cases, higher *CD274* transcript levels were not significantly associated with improved survival (*p* = 0.053), and *CD274* expression was not an independent prognostic factor in multivariable analysis [17]. In addition, in a TCGA-based analysis of 1016 NSCLC cases, high *CD274* mRNA expression was associated with worse OS in univariable analysis, although this association was not statistically significant after multivariable adjustment (adjusted HR 1.125, 95% CI 0.917–1.379; *p* = 0.26) [32]. The clinical relevance of *CD274* may therefore vary according to the molecular background of the tumour and on the simultaneous activation of other mechanisms promoting immune escape or tumour progression. Nevertheless, several other retrospective studies found no significant association between *CD274* transcript expression and survival outcomes [20,30,31].

Prospective evidence is considerably more limited and does not resolve these inconsistencies. In a prospective cohort of primary untreated lung cancer specimens, *CD274* transcript expression was not significantly associated with overall survival (OS) or progression-free survival (PFS) [27].

The prognostic associations of immune checkpoint transcripts appear to depend on their broader molecular and immune context, although the incremental prognostic value of multimarker profiles over single-gene assessment has not been established. A high *PDCD1/CTLA4*-derived ICS was associated with longer OS and RFS [34], whereas high *CD274* expression combined with *IDO2* was associated with less favourable outcomes [29]. Complementary protein-level evidence also supports this concept: in a prospective cohort, an unfavourable tumour-infiltrating lymphocyte immunotype was characterised by concurrent expression of multiple inhibitory receptors, including PD-1, TIM-3, and LAG-3 [35]. Together, these findings suggest that similar levels of *CD274* or *PDCD1* expression may have different clinical implications depending on the broader immune and molecular context [29,34,35]. 

The strength of the prognostic evidence is limited by substantial analytical heterogeneity. Across studies, RNA-expression cut-offs were derived using different approaches, multivariable adjustment was inconsistent, and independent validation was uncommon. Consequently, even statistically significant associations should be interpreted cautiously, particularly when thresholds were derived within the same cohort in which survival was subsequently evaluated. Studies evaluating the prognostic significance of *CD274* and *PDCD1* in NSCLC and their analytical details are summarised in Table 1.

### 5.2. Treatment-Associated Outcomes 

The treatment-associated significance of checkpoint-gene expression should be distinguished from its general prognostic value. Available evidence is derived predominantly from retrospective treatment-specific cohorts. In patients receiving pembrolizumab monotherapy, high *CD274* expression determined by RNA sequencing was associated with a higher response rate, longer PFS, and longer OS [26]. Patients whose tumours were classified as PD-L1-high by both RNA sequencing and immunohistochemistry demonstrated the highest response rate; however, this subgroup association does not by itself establish incremental predictive value of combined RNA- and protein-based assessment over either method alone [26].

Similar findings were observed in another retrospective cohort of patients with NSCLC treated with nivolumab, in which high *CD274* mRNA expression was associated with longer PFS [32]. In the RNA-seq-evaluable cohort (*n* = 43), high *CD274* mRNA expression was associated with longer PFS in univariable analysis (HR 0.332, 95% CI 0.140–0.788; *p* = 0.009) and remained significantly associated with PFS after adjustment for age, sex, WHO performance status, and histology (adjusted HR 0.225, 95% CI 0.079–0.638; *p* = 0.005) [32].

Complementary protein-level evidence indicates that the relationship between PD-L1 expression and treatment outcome may also depend on the therapeutic regimen and the cellular compartment assessed. In a retrospective cohort receiving pembrolizumab-based chemoimmunotherapy, high tumour-cell PD-L1 expression assessed by IHC was associated with longer PFS, whereas immune-cell PD-L1 expression was not informative. Conversely, among patients receiving nivolumab plus ipilimumab with or without platinum-doublet chemotherapy, high immune-cell PD-L1 expression was associated with longer PFS, while tumour-cell PD-L1 expression showed no significant association [23].

In contrast, in a small retrospective cohort of patients with *EGFR*-mutated lung adenocarcinoma, intratumoral *CD274* mRNA expression was associated with shorter PFS during gefitinib treatment but was not significantly associated with OS [33]. Prospective evidence is more limited; one prospective study of a mixed lung-cancer cohort (126 NSCLC and 15 SCLC) found no significant relationship between *CD274* mRNA expression and response to chemotherapy, predominantly paclitaxel- or gemcitabine combined with carboplatin-based regimens. Separate NSCLC-specific outcome analyses were not reported [27]. 

Fernandez et al. reported in a retrospective cohort of 122 patients with advanced NSCLC that high *CD274* mRNA expression, defined using the cohort median, was associated with greater long-term benefit at 24 months and longer OS [36]. The authors assessed long-term benefit at 12 and 24 months, although the operational response or progression criteria defining this endpoint were not further specified in the main report. Using an optimised cutoff of dCt + 10 = 7.1, low *CD274* expression showed a negative predictive value (NPV) of 0.92 for absence of long-term benefit at 24 months. The handling of patients with insufficient follow-up was not explicitly described. This cutoff was derived within the same retrospective cohort and was not independently validated.

*CD274* transcript expression may provide treatment-associated information in selected clinical settings, particularly in selected immune checkpoint inhibitor-treated cohorts. However, the available evidence is largely retrospective and treatment-specific, and associations with outcome vary according to therapeutic regimen, molecular background, immune-cell composition, and the method used to assess PD-L1. Consequently, current data are insufficient to establish *CD274* or *PDCD1* transcript expression as validated stand-alone predictive biomarkers. Multimarker profiles and combined RNA–protein assessment warrant further prospective evaluation, preferably in controlled cohorts allowing assessment of biomarker-by-treatment interactions [23,26,32,33].

Treatment-associated findings should likewise be interpreted cautiously. Most were derived from retrospective treatment-specific cohorts or relatively small analytical subsets, with heterogeneous treatment regimens, RNA-expression thresholds, and multivariable adjustment strategies. The limited use of independent validation and the general absence of biomarker-by-treatment interaction testing restrict the extent to which these associations can be considered predictive or generalisable. Studies evaluating treatment-associated outcomes and the potential predictive significance of *CD274* transcript expression, complementary PD-L1 protein evidence in NSCLC and their key analytical details are summarised in Table 2.

## 6. Methodological Challenges in Transcriptomic PD-1/PD-L1 Assessment

Despite its potential clinical value, transcriptomic assessment of the PD-1/PD-L1 axis is affected by several methodological factors that may contribute to the heterogeneity of published findings.

### 6.1. Biological Framework for Interpreting CD274 RNA–Protein Discordance

The heterogeneous clinical associations of *CD274* expression may be interpreted within a mechanism-to-measurement framework rather than as evidence for a single biological effect. One important source of heterogeneity arises from the cellular origin of the measured transcript. Bulk-tissue RNA reflects combined contributions from tumour, immune, and stromal cells, whereas PD-L1 IHC may evaluate tumour and immune compartments separately. Differences in immune-cell infiltration and compartment-specific PD-L1 expression therefore provide indirect support for the possibility that similar bulk *CD274* levels may represent biologically different tumour microenvironments [18,23]. Spatial heterogeneity and differences between primary and metastatic sites may further modify this relationship, although much of the supporting evidence is protein-based and should therefore be considered contextual rather than direct evidence for RNA heterogeneity [37]. Future studies using single-cell RNA sequencing and spatial transcriptomic approaches may help disentangle tumour-, immune-, and stromal-cell contributions to *CD274* expression and define its spatial distribution within the tumour microenvironment, thereby addressing some of the interpretative limitations of bulk-tissue RNA measurements.

Treatment exposure introduces an additional dynamic component. Chemotherapy has been associated with changes in broader immune-gene expression, although direct changes in *CD274* or *PDCD1* were not demonstrated [38]. Post-transcriptional, translational, and post-translational regulation may also contribute to RNA–protein discordance, but these mechanisms were not directly evaluated by most studies included in this review and should therefore be regarded as plausible rather than established explanations. Taken together, the available evidence supports several non-mutually exclusive mechanisms that may contribute to discordant RNA–protein relationships and opposing clinical associations; however, current studies do not allow their relative contributions to be resolved in individual NSCLC cohorts. Proposed biological framework for interpreting *CD274* transcript expression and RNA–protein discordance in NSCLC is presented in Table 3.

### 6.2. Analytical and Methodological Sources of Heterogeneity

Studies comparing *CD274* mRNA with PD-L1 protein expression used different analytical measures, including continuous correlations, categorical agreement, and probabilities of IHC classification [39,40,41,42]. For example, Sheffield et al. compared *CD274* RNA ISH with tumour-cell PD-L1 IHC using four antibody clones (SP142, E1L3N, 28-8, and RBT-PDL1) in 80 non-squamous NSCLC cases and reported clone-dependent RNA–protein relationships [39]. Moreover, PD-L1 IHC status differed between matched primary tumours and nodal metastases in 22% of cases, illustrating both assay- and sampling-site-dependent heterogeneity. 

Importantly, agreement between RNA- and protein-based measurements appears to be asymmetric. Low *CD274* RNA expression was generally associated with a high probability of a negative PD-L1 IHC result, although this probability varied according to the IHC assay and positivity threshold [40,42]. In the CLOVER study, the probability of IHC negativity among RNA-low/negative cases ranged from 70% to 97% depending on the assay and threshold; notably, at the 22C3 TPS ≥1% positivity threshold, only 70% of RNA-low/negative cases were IHC-negative, indicating that approximately 30% were IHC-positive under this definition. Conversely, among RNA-high cases, only 9–45% were classified as IHC-positive [40]. Similar findings were reported by Venina et al. [42]. These results indicate that RNA-based classification is assay- and threshold-specific rather than uniformly reliable across IHC definitions. In selected assay–threshold combinations, low *CD274* expression may therefore help identify tumours likely to be PD-L1-negative by IHC, whereas high RNA expression does not reliably confirm IHC positivity. Importantly, this refers only to classification against PD-L1 IHC status and should not be interpreted as evidence that low *CD274* expression can exclude clinical benefit from immunotherapy. Current evidence therefore relates primarily to classification against PD-L1 IHC status and does not establish *CD274* RNA assessment as a replacement for IHC, a means of excluding patients from immunotherapy, or a validated component of a multimarker treatment-selection model. These estimates are assay- and population-dependent and should not be interpreted as universally applicable predictive values. Importantly, these IHC-classification probabilities are distinct from the treatment-outcome NPV reported by Fernandez et al. [36], which is discussed separately in Section 5.2. Technical characteristics and classification metrics of the studies underlying these observations are summarised in Table 4. 

Importantly, the statistical measures used across studies address different analytical questions and should not be interpreted interchangeably. Correlation coefficients quantify the relationship between continuous RNA and protein measurements, whereas κ statistics assess agreement after categorisation according to predefined thresholds. Similarly, conditional probabilities and predictive values describe classification performance at specific RNA and IHC cut-offs and may depend on the distribution of IHC categories in the investigated population. These measures therefore provide complementary, rather than equivalent, information and do not by themselves establish clinical utility.

Technical aspects of reverse transcription quantitative polymerase chain reaction (RT-qPCR) may represent an additional source of variability. Søes et al. demonstrated that commonly used reference genes, including *ACTB*, *GAPDH*, and 18S rRNA, were insufficiently stable in several types of primary and metastatic NSCLC tissue [43]. The most stable combinations were *ACTR3* and *PUM1* or *TBP* and *PUM1* in lung tissue and *ACTR3* and *SF3A1* or *PUM1* and *IPO8* in lymph node metastases [43]. Reference genes should therefore be validated for the specific tissue type rather than selected solely on the basis of their widespread use.

Tissue preservation may also affect RNA quality and assay performance. In a single-patient paired comparison, Jacobson et al. showed that interpretable gene-expression profiles could be obtained from long-term preserved FFPE NSCLC tissue and showed good agreement with matched fresh-frozen material. However, this evidence was based on a single paired specimen from one patient, analytical sensitivity was lower in FFPE material, and *CD274* and *PDCD1* were not directly assessed [44]. Therefore, equivalence between FFPE and fresh-frozen tissue should not be assumed, as assay performance may depend on fixation conditions, RNA quality, storage duration, amplicon length, and the analytical platform.

The principal RNA-based platforms used in the reviewed studies also differ in their analytical characteristics. RT-qPCR provides targeted and sensitive quantification but is dependent on RNA quality, reference-gene selection, and pre-analytical processing [43,44]. Bulk RNA sequencing enables broader transcriptomic profiling but integrates signals from tumour, immune, and stromal compartments and therefore lacks cellular resolution. Targeted expression panels permit multiplexed measurement of predefined transcripts but remain dependent on platform-specific normalisation and thresholding. In contrast, RNA in situ approaches preserve spatial and cellular information but generally provide more targeted and less comprehensive expression assessment [41]. Consequently, results and cut-offs derived using different platforms should not be assumed to be directly transferable.

## 7. Limitations of the Review

This review has several limitations. First, as a narrative review, it used an exploratory rather than systematic study-selection approach, and no formal evidence-grading or risk-of-bias framework was applied. The literature search was limited to PubMed and English-language publications, and relevant studies may therefore have been missed. Publication and selective-outcome bias could not be formally assessed.

Interpretation was further limited by substantial heterogeneity among the included studies, including differences in assay platforms, expression thresholds, study populations, and clinical endpoints. Some secondary analyses were derived from the same public transcriptomic datasets and may therefore include overlapping patient populations, limiting their independence. Protein-only studies were included where relevant to provide biological or methodological context, but these were not considered direct evidence of transcript-based biomarker performance. The prognostic and treatment-associated literature was also heterogeneous with respect to RNA-expression cut-offs, covariate adjustment, analysis-specific sample sizes, and validation strategies. Several reported associations were derived from internally defined thresholds or small retrospective subgroups, limiting direct comparison and external generalisability. Finally, most treatment-associated studies were retrospective or lacked an appropriate comparator group, preventing reliable assessment of biomarker-by-treatment interactions and therefore limiting conclusions regarding predictive utility.

## 8. Conclusions and Recommendations for Future Studies

*CD274* transcript expression may provide clinically relevant information in NSCLC, particularly as a complement to protein-based PD-L1 assessment, whereas the available evidence for *PDCD1* is more limited and is derived largely from co-expression analyses rather than independently validated single-gene performance. In selected assay- and threshold-specific settings, low *CD274* expression may help identify tumours likely to be PD-L1-negative by immunohistochemistry [40,42]. However, current findings remain inconsistent, and neither marker can yet be considered a validated stand-alone prognostic or predictive biomarker. Current evidence demonstrates associations with clinical outcomes but does not establish predictive treatment interactions or incremental clinical utility beyond established PD-L1 immunohistochemistry. This heterogeneity appears to result from both biological differences between tumours and substantial methodological variability.

Future studies should analyse lung adenocarcinoma and squamous cell carcinoma separately and account for tumour stage, molecular alterations, immune-cell infiltration, and treatment exposure. The potential value of low *CD274* expression for identifying tumours likely to be PD-L1-negative by IHC should be evaluated using predefined cut-offs and independent validation cohorts. Multigene transcript profiles combining *CD274* or *PDCD1* with other immune or molecular markers, including *CTLA4* and *IDO2*, should be compared directly with single-gene models [29,34]. Protein-level findings involving TIM-3 and LAG-3 may additionally motivate future transcript-based hypotheses but do not constitute direct validation of such RNA panels [35]. However, the evidence supporting these multigene profiles remains limited and heterogeneous, and their clinical utility has not yet been established prospectively.

Future biomarker studies may also benefit from integrating transcriptomic data with complementary molecular layers, including proteomic and epigenomic information, potentially supported by AI-assisted multi-omics approaches to capture the complex regulation of immune-checkpoint expression more comprehensively [45].

Methodological standardisation remains essential. RT-qPCR studies should validate reference genes for each tissue type, particularly when comparing primary lung tumours with lymph node metastases [43]. Future research should also report tissue composition, sampling site, specimen preservation, and RNA-assessment procedures in sufficient detail. Larger prospective cohorts and external validation will be required to determine whether *CD274* and *PDCD1* transcript expression provides clinically useful information beyond established PD-L1 immunohistochemistry. Establishing predictive utility will additionally require demonstration of a biomarker-by-treatment interaction, ideally in randomised or appropriately controlled cohorts.

## Figures and Tables

**Table 1:** Summary of studies evaluating the prognostic significance of *CD274*, *PDCD1*, and related transcriptomic profiles in NSCLC.

Study	Study Design	Cohort	Main Prognostic Findings	Key Analytical Details
Usó et al., 2016 [34]	Retrospective cohort study	Surgically resected NSCLC, *n* = 178 (Stage I–IIIA)	A high immune checkpoint score (ICS) based on *PDCD1* and *CTLA4* expression was independently associated with longer OS (HR 0.308, 95% CI 0.156–0.609; *p* = 0.001) and RFS (HR 0.527, 95% CI 0.298–0.933; *p* = 0.028).	ICS derived from *PDCD1* and *CTLA4* expression within the study cohort and dichotomised at the cohort median. Multivariable analysis included lymph-node involvement, *KRAS* status, cluster classification, FOXP3, CD4+, CD127, IL23, LGALS2, and ICS. Median follow-up: 81.23 months (range 1–113). No independent validation cohort was reported.
Ludovini et al., 2021 [29]	Retrospective cohort study	Radically resected NSCLC *n* = 191 (Stage I–III)	High co-expression of *CD274* and *IDO2* was significantly associated with worse OS in Cox regression (HR 1.98; 95% CI 1.02–3.83, *p* = 0.04)	Expression cutoff: ≥4-fold vs. matched normal lung; outcome-derived with internal bootstrap optimisation. Multivariable Cox analysis adjusted for stage. Median follow-up: 66 months; no external validation.
Öjlert et al., 2019 [19]	Retrospective cohort study	Surgically resected NSCLC, *n* = 399 (Stage I–IV)	Increasing *CD274* expression was associated with better prognosis in the secretory subtype (*n* = 48), whereas no significant association was observed in the overall SCC cohort.	*CD274* analysed continuously per 1-standard deviation increase; stage-adjusted Cox model. Overall SCC: HR 1.08, 95% CI 0.85–1.37. No RNA cutoff or external validation cohort.
Kim et al., 2019 [17]	Retrospective cohort study	Surgically resected AD and SCC specimens from National biobank of Korea *n* = 71 (paired RNA–protein analysis *n* = 8)	Higher *CD274* expression showed a nonsignificant trend toward better OS (*p* = 0.053) and was not significantly associated with disease-free survival (*p* = 0.073); *CD274* was not an independent prognostic factor in multivariable analysis.	Tumour/normal expression ratio dichotomised at the cohort mean (3.72); OS *n* = 70, disease-free survival (DFS) *n* = 68. Median follow-up: 76.7 months. Multivariable analysis performed, but HR/95% CI for PD-L1 were not reported; no external validation cohort.
Kim et al., 2018 [20]	Retrospective cohort study	NSCLC samples *n* = 687	In early-stage adenocarcinoma, PD-L1 protein expression was associated with shorter OS, whereas *CD274* mRNA showed no prognostic significance	RNAscope-based binary cutoff. No HR/95% CI for *CD274* mRNA or external validation cohort reported.
Sepesi et al., 2018 [30]	Bioinformatic analysis of a public transcriptomic dataset	TCGA NSCLC cases, *n* = 985	*CD274* expression showed no prognostic value for OS	TCGA-based cohort; multiple *CD274* mRNA cutoffs tested, including the median, with no significant OS association. No external validation.
Kumar et al., 2022 [27]	Prospective cohort study	Primary untreated lung cancer cohort: *n* = 141 evaluable (126 NSCLC, 15 SCLC); additional surgical NSCLC specimens *n* = 32 for IHC assessment.	*CD274* mRNA expression was not associated with chemotherapy response, OS, or PFS.	Prospective mixed lung-cancer cohort (126 NSCLC, 15 SCLC); RT-qPCR-based mRNA assessment. Outcome analyses were not reported separately for NSCLC; no external validation cohort.
Sasaki et al., 2013 [31]	Observational cohort study	Surgically resected NSCLC *n* = 123	*PD-L1 mRNA/β-actin* expression was not significantly associated with survival (*p* = 0.2336)	Tumor/Normal expression ratio ≥1 vs. <1; Kaplan–Meier/log-rank analysis (*n* = 123). HR/95% CI and multivariable survival analysis not reported; no external validation.
Fumet et al., 2018 [32]	Retrospective cohort study	TCGA NSCLC *n* = 1016	High *CD274* expression was associated with shorter OS (45.21 vs. 54.30 months; univariable HR 1.214, 95% CI 1.000–1.481; *p* = 0.05). In the multivariable model including clinical covariates and *CD274*, the association was not statistically significant (adjusted HR 1.125, 95% CI 0.917–1.379; *p* = 0.26).	RSEM-derived RNA-seq expression normalised using variance-stabilising transformation (DESeq2); outcome-optimised Cutoff Finder threshold; numerical *CD274* cutoff value was not reported; multivariable Cox analysis adjusted for age, sex, stage, and histology. Internal bootstrap validation; no external prognostic validation.
Velcheti et al., 2014 [24]	Retrospective cohort study	NSCLC samples *n* = 544 (Greek cohort: *n* = 340, RNA-evaluable *n* = 314; Yale cohort: *n* = 204, RNA-evaluable *n* = 173).	High *CD274* expression was associated with longer OS in both the Greek (HR 0.64, 95% CI 0.46–0.90) and Yale cohorts (HR 0.50, 95% CI 0.30–0.82) in univariable analysis; the association remained significant after multivariable adjustment in the Yale cohort but not in the Greek cohort.	Assay-based DapB detection threshold; Cox models adjusted for stage, histology, and TILs. Independent two-cohort validation, although multivariable significance was retained only in the Yale cohort.

DFS, disease-free survival.

**Table 2:** Summary of studies evaluating treatment-associated outcomes of *CD274* transcript expression and complementary PD-L1 protein evidence in NSCLC.

Study	Study Design	Cohort	Treatment Cohort	Main Treatment-Associated Findings	Key Analytical Details
Kumar et al. [27]	Prospective cohort	Primary untreated lung cancer cohort: *n* = 141 evaluable (126 NSCLC, 15 SCLC); additional surgical NSCLC specimens *n* = 32 for IHC assessment.	Paclitaxel- or gemcitabine combined with carboplatin-based chemotherapy	In the mixed lung-cancer cohort, *CD274* mRNA expression was not associated with chemotherapy response, OS, or PFS.	Prospective mixed lung-cancer cohort (126 NSCLC, 15 SCLC); RT-qPCR-based mRNA assessment. Outcome analyses were not reported separately for NSCLC; no external validation cohort.
Isobe et al. [33]	Retrospective cohort	Surgically resected recurrent postoperative *EGFR*-mutant lung adenocarcinoma *n* = 33	Gefitinib	Intratumoral *CD274* mRNA expression was associated with shorter PFS (HR 2.95, 95% CI 1.27–6.87; *p* = 0.012) but not OS (1291 vs. 1511 days; *p* = 0.24).	Detection-based binary classification (detectable vs. undetectable intratumoral mRNA); multivariable Cox model included pleural and bone metastases and *CD274* expression. Small single-centre cohort (*n* = 33)
Nesline et al. [26]	Retrospective cohort	Pembrolizumab-treated NSCLC cohort *n* = 102 (response-evaluable *n* = 74)	Pembrolizumab	High *CD274* RNA expression was associated with higher response rate (65% vs. 18%; *p* < 0.001), longer PFS (19.2 vs. 4.0 months; adjusted HR 0.62, 95% CI 0.42–0.82), and longer OS (24.3 vs. 5.4 months; adjusted HR 0.45, 95% CI 0.24–0.84), during pembrolizumab monotherapy. Combined RNA/IHC-high status identified the highest PFS (HR 0.32, 95% CI 0.14–0.77 vs. IHC-low/RNA-not-high).	Prespecified clinical RNA-seq cutoff ≥75th percentile rank, supported by ROC analysis. Multivariable Cox models adjusted for age, sex, histology, smoking, weighted ECOG status, and treatment line. Outcome cohort *n* = 102; response-evaluable *n* = 74. No external outcome-validation cohort.
Fumet et al. [32]	Retrospective cohort	Primary nivolumab cohort *n* = 85 (RNA-seq-evaluable *n* = 43); independent anti-PD-1 validation cohort *n* = 44.	Nivolumab in the primary cohort; nivolumab or pembrolizumab in the validation cohort.	High *CD274* mRNA expression was associated with longer PFS in the nivolumab-treated RNA-evaluable cohort (*n* = 43; median PFS not reached vs. 2.8 months; univariable HR 0.332, 95% CI 0.140–0.788; *p* = 0.009) and remained significant in multivariable analysis (adjusted HR 0.225, 95% CI 0.079–0.638; *p* = 0.005). The association was reproduced in the independent anti-PD-1 validation cohort (*n* = 44; pembrolizumab in first line or nivolumab in later lines).	RNA-seq expression based on RSEM counts normalised by variance-stabilising transformation (DESeq2); outcome-optimised Cutoff Finder threshold. Numerical *CD274* cutoff value was not reported. Cutoff Finder was also used in the validation cohort; use of an unchanged primary-cohort cutoff was not reported. Multivariable Cox model adjusted for age, sex, WHO performance status, and histology.
Fernandez et al., 2022 [36]	Retrospective cohort study	122 NSCLC samples (Stage III-IV) (Atezolizumab *n* = 14; atezolizumab + chemotherapy *n* = 2; durvalumab + tremelimumab *n* = 2; nivolumab *n* = 56; nivolumab + ipilimumab *n* = 4; pembrolizumab *n* = 44)	Atezolizumab or atezolizumab + chemotherapy, or durvalumab + tremelimumab, or nivolumab, or nivolumab + ipilimumab, or pembrolizumab	High *CD274* and *PDCD1LG2* mRNA expression was associated with greater long-term benefit at 24 months (*CD274*: *p* = 0.047) and longer OS (*CD274*: *p* = 0.048). Using a separately optimised cutoff, low *CD274* expression showed an NPV of 0.92 for absence of long-term benefit.	Median cutoff (dCt + 10 = 6.6) for survival and clinical-benefit analyses; separately optimised cutoff (dCt + 10 = 7.1; cutpointr) for positive predictive value/negative predictive value analysis. NPV = 0.92 for absence of 24-month long-term benefit. The operational definition of long-term benefit beyond the assessment time point and handling of insufficient follow-up were not explicitly reported. Analysis-specific denominator and 95% CI for the NPV were not reported. No independent validation.
Miyakoshi et al. [23]	Retrospective cohort	NSCLC patients treated with pembrolizumab-based chemoimmunotherapy (*n* = 137) or nivolumab + ipilimumab-based therapy (*n* = 61); total *n* = 198.	Pembrolizumab + chemotherapy; nivolumab + ipilimumab ± platinum-doublet chemotherapy	Complementary IHC-based evidence: In the pembrolizumab-based cohort (*n* = 137), high tumour-cell TPS was associated with longer PFS (HR 0.59, 95% CI 0.38–0.92), whereas IC score was not. In the nivolumab + ipilimumab cohort (*n* = 61), high IC score was associated with longer PFS (HR 0.53, 95% CI 0.28–0.96), whereas TPS was not. Among patients with low TPS/high IC scores, nivolumab + ipilimumab showed a greater 24-month restricted mean survival time (RMST) than pembrolizumab-based chemoimmunotherapy (RMST ratio 1.50, 95% CI 1.00–2.32).	Prespecified IHC cutoffs: TPS ≥50%, IC score ≥1. Low-TPS/high-IC treatment comparison required RMST analysis because the proportional-hazards assumption was violated. Treatment-outcome analyses were IHC-based; RNA-seq was performed separately in a treatment-naïve cohort (*n* = 152). No independent prospective validation.

ICs, immune cells; NPV, negative predictive value; RMST, restricted mean survival time; ROC, receiver operating characteristic.

**Table 3:** Proposed biological framework for interpreting *CD274* transcript expression and RNA–protein discordance in NSCLC.

Biological/Clinical Context	Potential Influence on *CD274* Assessment	Evidence in the Reviewed Literature	Interpretation
Cellular composition of bulk tissue	Bulk RNA integrates transcript contributions from tumour, immune, and stromal cells	Differences in immune-cell infiltration and compartment-specific PD-L1 expression [18,23]	Indirect evidence that similar bulk *CD274* levels may represent different cellular contexts
Spatial and sampling heterogeneity	RNA/protein measurements may vary according to sampled region or anatomical site	Spatial PD-L1 heterogeneity and primary–metastatic discordance [37]	Contextual protein-level evidence; relevance to RNA measurement is plausible but indirect
Treatment-related dynamics	Therapy may modify the immune-transcriptional environment	Chemotherapy-associated changes in broader immune-gene expression [38]	Indirect evidence; direct treatment-induced changes in *CD274*/*PDCD1* were not demonstrated
Post-transcriptional/translational regulation	mRNA abundance may not directly correspond to protein abundance	Variable RNA–protein concordance across studies [39,40,41,42]	Plausible contributor to discordance, but not directly resolved by the available NSCLC evidence

**Table 4:** Technical characteristics of studies evaluating RNA-based classification of PD-L1 IHC status.

Study	Study Population	RNA Assay and Cutoff	PD-L1 IHC Assay and Cutoffs	Relevant Classification Result
Tsimafeyeu et al. (CLOVER) [40]	NSCLC (IHC *n* = 473, RNA-evaluable *n* = 437, RNA-high *n* = 187 and RNA-low/absent *n* = 250). Assay-specific paired denominators underlying the conditional percentages were not reported.	TaqMan RT-PCR; *SDHA* reference; ΔCt = 2 used as the threshold between high and low/absent RNA expression. The exact ΔCt calculation convention and direction relative to the cutoff were not reported	**22C3:** TPS ≥50% (1L), ≥1% (2L); **SP142:** TC/IC ≥5% (1L), TC ≥50% or IC ≥10% (2L); **SP263:** TC ≥25%	Given low/negative RNA expression, the probability of IHC negativity was 91%/97% for SP142, 91%/91% for SP263, and 97%/70% for 22C3 (first-/second-line cutoffs, respectively). Given high RNA expression, corresponding probabilities of IHC positivity were 27%/9%, 26%/26%, and 17%/45%, respectively.
Venina et al. [42]	Non-squamous NSCLC (*n* = 167)	PCR-based RNA assessment; low/moderate/high categories defined using arbitrarily selected 20th and 70th percentile cutoffs of RNA expression in the analysed cohort (*n* = 167). The study reported 52 RNA-low cases for the 1% IHC comparisons; the relationship between this denominator and the percentile-based categorisation was not explicitly explained.	**22C3 and SP263:** TC thresholds 1% and 50%; **SP142:** TC/IC-based scoring	Among RNA-low cases, 98% (51/52) were 22C3-negative and 92% (48/52) were SP263-negative at the 1% TC threshold.

1L, first-line; 2L, second-line; TCs, tumour cells; ICs, immune cells; TPS, tumour proportion score. Reported percentages represent conditional probabilities of an IHC result given the RNA-expression category [e.g., P(IHC-negative|RNA-low)] and should not be interpreted as sensitivity of RNA testing for detecting IHC-positive cases or as sensitivity for predicting treatment benefit.

## Data Availability

No new data were created or analyzed in this study. Data sharing is not applicable to this article. The original histopathological images presented in Figure 1 are derived from anonymised archival material and are retained by the Department of Pathomorphology, Medical University of Silesia in Katowice. Access may be subject to institutional restrictions.

## Supplementary Materials

The following supporting information can be downloaded at https://www.mdpi.com/article/10.3390/biomedicines14102318/s1, Table S1: Methodological characteristics and main findings of studies evaluating *CD274* and *PDCD1* transcript expression in NSCLC.

## Abbreviations

The following abbreviations are used in this manuscript:

AD	Adenocarcinoma
EBUS-TBNA	Endobronchial ultrasound-guided transbronchial needle aspiration
FFPE	Formalin-Fixed Paraffin-Embedded
ICIs	Immune checkpoint inhibitors
ICS	Immune checkpoint score
IHC	Immunohistochemistry
NSCLC	Non-small cell lung carcinoma
OS	Overall survival
PFS	Progression-free survival
PI3K	Phosphatidylinositol 3-kinase
RFS	Relapse-free survival
RMST	Restricted mean survival time
RT-qPCR	Reverse transcription quantitative polymerase chain reaction
SCC	Squamous cell carcinoma
TCGA	The Cancer Genome Atlas
TPS	Tumour proportion score
