# Supplementary material for: Transcriptomic Assessment of PD-L1 (CD274) in NSCLC: Biological Significance, Clinical Relevance, and Methodological Challenges
Source: Biomedicines. 2026 Oct 12;14(10):2318. doi: 10.3390/biomedicines14102318 (PMC13701400; doi:10.3390/biomedicines14102318)
Supplement: Supplementary file 1 [file biomedicines-14-02318-s001.zip › biomedicines-4536907-supplementary.pdf]

Table S1. Methodological characteristics and main findings of studies evaluating *CD274* and *PDCD1* transcript expression in NSCLC.

Authors	Study Design	Cohort	Specimen and Analytical Methods	Main Findings	Key Limitations	Evidence Role
Liebler et al., 2020 [13]	Retrospective design	Immune checkpoint inhibitor naïve NSCLC samples $n = 46$	Tumour tissues preserved by FFPE, RNA-seq, MS	PD-1 showed weak RNA–protein correlation, whereas PD-L1 mRNA correlated substantially with protein abundance measured by mass spectrometry. PD-L1 measurements by MS and IHC showed only weak concordance. LAG-3 and TIM-3 were detectable at both RNA and protein levels, but their transcript and protein abundances were not significantly correlated.	Small cohort, retrospective design	Direct transcript-level evidence

Authors	Study Design	Cohort	Specimen and Analytical Methods	Main Findings	Key Limitations	Evidence Role
Kim et al., 2019 [17]	Retrospective cohort study	Surgically resected AD and SCC specimens from National biobank of Korea *n* = 71, paired RNA–protein analysis *n* = 8	IHC, qPCR, tissue microarray	*CD274* mRNA expression correlated positively with PD-L1 protein levels and was approximately threefold higher in tumour than in matched non-neoplastic tissue. Higher *CD274* expression was not significantly associated with survival ($p = 0.053$), histological subtype, or tumour stage.	Retrospective design; small cohort (*n* = 71); RNA–protein concordance was assessed in only eight cases; *CD274* expression was not an independent prognostic factor in multivariable analysis.	Direct transcript-level evidence

Authors	Study Design	Cohort	Specimen and Analytical Methods	Main Findings	Key Limitations	Evidence Role
Cheng et al., 2023 [18]	Transcriptomic cohort study	67 early-stage NSCLC (Stage I-II)	RNA-seq	Tumour and adjacent non-neoplastic tissues showed markedly different immune transcriptomic profiles. Adjacent non-neoplastic samples had significantly higher inferred immune infiltration, and *CD274* expression was lower in tumour than in adjacent non-neoplastic tissue.	Small early-stage cohort; analyses focused on the broader immune microenvironment rather than *CD274* biomarker performance;	Direct transcript-level evidence

Öjlert et al., 2019 [19]	Retrospective cohort study	Surgically resected NSCLC, *n* = 399 (Stage I-IV)	Tumour tissues were frozen or FFPE, Microarray, RNA-seq, IHC	*CD274* mRNA expression correlated positively with PD-L1 protein levels and immune-cell infiltration, particularly macrophages, NK cells, and cytotoxic T cells. Its association with the immune microenvironment was stronger than that of PD-L1 protein expression. Within SCC expression subtypes, increasing *CD274* expression was associated with better prognosis in the secretory subtype (*n* = 48), whereas the association was reversed in the classical subtype (*n* = 85). No significant association was observed in the overall	Retrospective design; single-centre cohort	Direct transcript-level evidence

Authors	Study Design	Cohort	Specimen and Analytical Methods	Main Findings	Key Limitations	Evidence Role
				SCC cohort (HR 1.08, 95% CI 0.85–1.37).		
Kim et al., 2018 [20]	Retrospective cohort study	NSCLC samples *n* = 687 (adenocarcinoma *n* = 476; squamous cell carcinoma *n* = 211)	Tumour tissues preserved by FFPE, TMA, IHC, in situ hybridization, PCR/DNA sequencing for *EGFR* and *KRAS* mutation analysis	PD-L1 protein and *CD274* mRNA expression were strongly correlated and were associated with histological differentiation within adenocarcinoma and with *EGFR* status, with lower expression in *EGFR*-mutated tumours. Neither protein nor mRNA expression was associated with survival in the overall cohort. In early-stage adenocarcinoma, PD-L1 protein expression was associated with shorter OS, whereas *CD274* mRNA showed no prognostic significance.	Lack of response data to anti-PD-1/PD-L1 therapy	Direct transcript-level evidence

Authors	Study Design	Cohort	Specimen and Analytical Methods	Main Findings	Key Limitations	Evidence Role
Alwithenani et al., 2021 [21]	Retrospective cohort study	Surgically resected NSCLC samples n = 851, RT-qPCR-evaluable subset n = 49	FFPE tissue preservation, RT-qPCR, IHC	PD-L1 protein expression was associated with wild-type *EGFR* and vascular invasion. *CD274* mRNA levels showed a weak but significant correlation with PD-L1 protein expression by IHC, which slightly increased when both tumour and immune-cell PD-L1 were considered.	Single-centre surgical cohort; RT-qPCR performed only in a small subgroup (n = 49)	Direct transcript-level evidence
Wu et al., 2017 [22]	Retrospective design	Surgically resected lung adenocarcinoma n = 133	IHC, TMA, *in situ* hybridization	PD-L1 expression was detected in 16.5% of lung adenocarcinomas at the mRNA level and in 13.5% at the protein level. The two methods showed high concordance (κ = 0.824).	Single-centre surgical cohort	Direct transcript-level evidence

Authors	Study Design	Cohort	Specimen and Analytical Methods	Main Findings	Key Limitations	Evidence Role
Miyakoshi et al., 2025 [23]	Retrospective cohort study	NSCLC patients treated with pembrolizumab-based chemoimmunotherapy (*n* = 137) or nivolumab + ipilimumab-based therapy (*n* = 61); total *n* = 198.	WES and IHC in treatment cohorts; RNA-seq performed separately in a treatment-naïve cohort (*n* = 152).	In the pembrolizumab-based chemoimmunotherapy cohort, high PD-L1 tumour proportion score (TPS) was associated with longer PFS, whereas immune-cell PD-L1 expression was not. Conversely, in the nivolumab plus ipilimumab cohort, high immune-cell PD-L1 expression was associated with longer PFS, while TPS showed no significant association. *CD274* transcript expression was significantly lower in tumours with low TPS/high immune-cell PD-L1 expression than in those with high TPS.	The sample size of the nivolumab + ipilimumab cohort was relatively small, lack of experimental validation of immunosuppressive mechanisms mediated by regulatory T cells in P-II tumours (low TPS/high immune-cell PD-L1 expression).	Direct transcript-level evidence for *CD274* expression; contextual IHC-based treatment-outcome evidence.

Authors	Study Design	Cohort	Specimen and Analytical Methods	Main Findings	Key Limitations	Evidence Role
Velcheti et al., 2014 [24]	Retrospective cohort study	NSCLC samples *n* = 544 (Greek cohort: *n* = 340, RNA-evaluable *n* = 314; Yale cohort: *n* = 204, RNA-evaluable *n* = 173)	Tissues preserved by FFPE, IHC, TMA, In Situ Hybridization, QIF	PD-L1 mRNA and protein expression showed a positive but non-linear relationship. PD-L1 expression was higher in squamous cell carcinoma than in adenocarcinoma, and higher PD-L1 mRNA levels were significantly associated with longer OS.	Retrospective design, work was performed on TMAs	Direct transcript-level evidence

Authors	Study Design	Cohort	Specimen and Analytical Methods	Main Findings	Key Limitations	Evidence Role
Erber et al., 2017 [25]	Prospective cohort study	NSCLC tissues *n* = 22 (including 8 primary tumours, 5 metastatic lesions and 9 EBUS-TBNA)	FFPE tissue preservation, RT-qPCR, IHC	*CD274* mRNA showed excellent concordance with PD-L1 IHC using the 28-8 antibody in primary NSCLC tissues and EBUS-TBNA samples (κ = 0.85–1.0). However, concordance was poor in metastatic lesions (κ = 0.20), indicating that RNA–protein agreement may depend on the sampled tissue site.	Very small cohort (*n* = 22)	Direct transcript-level evidence

Authors	Study Design	Cohort	Specimen and Analytical Methods	Main Findings	Key Limitations	Evidence Role
Nesline et al., 2023 [26]	Retrospective cohort study	Overall NSCLC cohort *n* = 3270: RNA–IHC concordance cohort *n* = 3168; pembrolizumab monotherapy outcome cohort *n* = 102 (response-evaluable *n* = 74).	FFPE tissue preservation, RNA-seq, IHC	PD-L1 RNA-seq and IHC showed moderate concordance, with most discordant cases exhibiting low/negative IHC but high RNA expression. In a pembrolizumab-treated cohort, high *CD274* expression by RNA-seq was associated with higher response rates, longer PFS, and longer OS, whereas IHC alone was not significantly associated with outcomes. Patients classified as PD-L1-high by both methods showed the highest response rate.	Retrospective nature of the study	Direct transcript-level evidence

Authors	Study Design	Cohort	Specimen and Analytical Methods	Main Findings	Key Limitations	Evidence Role
Kumar et al., 2022 [27]	Prospective cohort study	Primary untreated lung cancer cohort: *n* = 141 evaluable (126 NSCLC, 15 SCLC); additional surgical NSCLC specimens *n* = 32 for IHC assessment.	RNAlater preserved frozen tissues, IHC, RT-qPCR	*CD274* mRNA expression was not associated with clinicopathological characteristics, driver mutations, chemotherapy response, OS, or PFS. PD-L1 TPS was likewise not associated with survival outcomes. However, *CD274* mRNA and PD-L1 protein expression showed a significant positive correlation.	Shorter follow-up; patients were not treated with ICI; Analyses of RNA–protein correlation, chemotherapy response, OS, and PFS were not reported separately for the NSCLC subgroup.	Direct transcript-level evidence
Simonsen et al., 2022 [28]	Retrospective design	42 NSCLC samples (32 included in the primary PD-L1 RNA–IHC analysis)	IHC, RNA-seq	Mean *CD274*/PD-L1 mRNA abundance was significantly lower in the IHC PD-L1-low group than in the PD-L1-high group, both in histological subgroups and in the pooled cohort.	Retrospective design, Small cohort, Heterogeneous specimen types; No validated RNA cutoff or external validation.	Direct transcript-level evidence

Authors	Study Design	Cohort	Specimen and Analytical Methods	Main Findings	Key Limitations	Evidence Role
Ludovini et al., 2021 [29]	Retrospective cohort study	Radically resected NSCLC *n* = 191 (Stage I-III)	Tumour tissues preserved in RNAlater and stored frozen; RT-qPCR	*PDCD1* and *CD274* transcript levels were significantly higher in patients with more advanced TNM stage and in those with squamous cell carcinoma. Cox regression analysis showed that high co-expression of *CD274* and *IDO2* was significantly associated with worse OS after adjustment for pathological stage (HR 1.98, 95% CI 1.02–3.83; p = 0.04).	Retrospective design; gene expression signatures are subject to sampling bias caused by intratumor genetic heterogeneity; patients were not treated with immune checkpoint inhibitors	Direct transcript-level evidence

Authors	Study Design	Cohort	Specimen and Analytical Methods	Main Findings	Key Limitations	Evidence Role
Sepesi et al., 2018 [30]	Bioinformatic analysis of a public transcriptomic dataset	TCGA NSCLC cases, *n* = 985	TCGA analysis	*CD274* mRNA expression was higher in lung cancer than in most other tumour and normal tissues and was significantly higher in squamous cell carcinoma than in adenocarcinoma. Expression was associated with pathological stage in squamous cell carcinoma and smoking status in adenocarcinoma but showed no prognostic value for OS.	Retrospective secondary analysis of a public dataset; lack of independent clinical and methodological validation.	Public-dataset analysis

**Authors**	**Study Design**	**Cohort**	**Specimen and Analytical Methods**	**Main Findings**	**Key Limitations**	**Evidence Role**
Sasaki et al., 2013 [31]	Observational cohort study	Surgically resected NSCLC n = 123	NSCLC tissues were stored frozen, RT-qPCR	*PD-L1 mRNA/β-actin* expression was not significantly associated with histological subtype, lymph node metastasis, or patient survival.	Lack of IHC PD-L1 expression validation	Direct transcript-level evidence

Fumet et al., 2018 [32]	Retrospective cohort study	TCGA NSCLC *n* = 1016; chemotherapy-treated cohort without immunotherapy *n* = 34; primary nivolumab cohort *n* = 85 (RNA-seq-evaluable *n* = 43); independent anti-PD-1 validation cohort *n* = 44 (pembrolizumab in first line or nivolumab in later lines).	Tissues were preserved by FFPE, IHC, RNA-seq, RSEM-derived expression values normalised using variance-stabilising transformation; outcome-optimised Cutoff Finder thresholds.	In the TCGA cohort, high *CD274* mRNA expression was associated with worse OS in univariable analysis (HR 1.214, 95% CI 1.000–1.481; p = 0.05), but the association was not statistically significant after multivariable adjustment (adjusted HR 1.125, 95% CI 0.917–1.379; p = 0.26). In the nivolumab-treated RNA-evaluable cohort (*n* = 43), high *CD274* expression was associated with longer PFS in univariable analysis (HR 0.332, 95% CI 0.140–0.788; p = 0.009) and remained significant after multivariable adjustment (HR 0.225, 95% CI 0.079–0.638; p =	Retrospective design; RNA-based treatment-outcome analysis was limited to 43 evaluable patients in the primary nivolumab cohort; the numerical *CD274* cutoff value was not reported. Cutoff Finder was also used in the validation cohort; use of an unchanged primary-cohort cutoff was not reported. No prospective external validation. The article text reports significance for *CD274* after bootstrap validation (p = 0.02); however, this is inconsistent	Direct transcript-level evidence; public-dataset analysis,

Authors	Study Design	Cohort	Specimen and Analytical Methods	Main Findings	Key Limitations	Evidence Role
				0.005). The association was reproduced in an independent anti-PD-1 validation cohort (*n* = 44).	with the numerical results presented in Supplementary Table 6.	
Isobe et al., 2018 [33]	Retrospective cohort study	Surgically resected recurrent postoperative *EGFR*-mutant lung adenocarcinoma *n* = 33	FFPE tissue preservation, RT-qPCR, IHC	Intratumoral *CD274* mRNA expression was positively correlated with *BIM* and inversely correlated with *VEGFA*. It was also associated with significantly shorter PFS during gefitinib therapy, but not with OS.	Single-centre study, small cohort (*n* = 33), cell line as control	Direct transcript-level evidence

Authors	Study Design	Cohort	Specimen and Analytical Methods	Main Findings	Key Limitations	Evidence Role
Usó et al., 2016 [34]	Retrospective cohort study	Surgically resected NSCLC, *n* = 178 (Stage I-IIIA)	Tumour tissues preserved in RNAlater and stored frozen; RT-qPCR	Hierarchical clustering identified a subgroup with higher expression of immunoregulatory genes and better survival. Separately, an immune checkpoint score (ICS) derived from weighted *PDCD1* and *CTLA4* expression and dichotomised at the cohort median was independently associated with longer OS (HR 0.308, 95% CI 0.156–0.609) and RFS (HR 0.527, 95% CI 0.298–0.933).	Retrospective design; single-centre cohort	Direct transcript-level evidence

**Authors**	**Study Design**	**Cohort**	**Specimen and Analytical Methods**	**Main Findings**	**Key Limitations**	**Evidence Role**
Federico et al., 2022 [35]	Prospective cohort study	NSCLC samples *n* = 150 (stage 0-IV)	RNA-seq, mIF, WES, Flow cytometry	Tumour-infiltrating T cells showed higher expression of inhibitory receptors, including PD-1, TIM-3, and LAG-3, than T cells from matched uninvolved lung tissue. A poor-prognosis TIL immunotype was characterised by enrichment of activated/proliferating T cells expressing multiple inhibitory receptors, including PD-1, TIM-3, and LAG-3.	Variable sample availability across analytical platforms	Protein-level contextual evidence

Authors	Study Design	Cohort	Specimen and Analytical Methods	Main Findings	Key Limitations	Evidence Role
Fernandez et al., 2022 [36]	Retrospective cohort study	122 NSCLC samples (Stage III-IV) (Atezolizumab *n* = 14; atezolizumab + chemotherapy *n* = 2; durvalumab + tremelimumab *n* = 2; nivolumab *n* = 56; nivolumab + ipilimumab *n* = 4; pembrolizumab *n* = 44)	RT-qPCR; PD-L1 IHC; median cutoff (dCt+10 = 6.6) for survival/long-term-benefit analyses; separate optimised cutoff (dCt+10 = 7.1; cutpointr) for PPV/NPV analysis; long-term benefit assessed at 12 and 24 months.	High *CD274* and *PDCD1LG2* mRNA expression was associated with greater long-term benefit at 24 months (*CD274*: p = 0.047) and longer OS (*CD274*: p = 0.048). Using the optimised cutoff (dCt+10 = 7.1), low *CD274* expression showed an NPV of 0.92 for absence of 24-month long-term benefit.	Retrospective single-centre study with heterogeneous ICI regimens; the operational definition of long-term benefit beyond the assessment time point and handling of patients with insufficient follow-up were not explicitly reported; analysis-specific denominator and 95% CI for the NPV were not reported; no independent validation.	Direct transcript-level evidence

Authors	Study Design	Cohort	Specimen and Analytical Methods	Main Findings	Key Limitations	Evidence Role
Haragan et al., 2019 [37]	Retrospective cohort study	107 resected primary NSCLCs and matched nodal metastases	IHC	Intratumoural heterogeneity of PD-L1 expression was common, and primary–nodal discordance was observed in 53% of cases; in 23%, the difference was sufficient to cross a clinically relevant PD-L1 cutoff.	Retrospective design; protein-level assessment only, without RNA analysis or treatment-response validation.	Protein-level contextual evidence

Authors	Study Design	Cohort	Specimen and Analytical Methods	Main Findings	Key Limitations	Evidence Role
Amrein et al., 2020 [38]	Prospective cohort study	*EGFR*-wild-type non-squamous NSCLC; 100 baseline biopsies and 33 progression re-biopsies, including 29 matched pairs	FFPE biopsies; laser-capture microdissection; NanoString nCounter 201-gene panel	A 47-gene immune co-expression module including *CD274* and *PDCD1* was identified and showed reduced overall expression after platinum-based chemotherapy. *CTLA4*, *LAG3*, *TNFRSF18*, *CD80*, and *FOXP3* were significantly decreased in matched samples, whereas *CD274* and *PDCD1* did not show significant individual changes.	Limited number of matched pre/post-treatment samples (n = 29); heterogeneous treatment regimens; no significant individual treatment-related change in *CD274* or *PDCD1*	Direct transcript-level evidence

Authors	Study Design	Cohort	Specimen and Analytical Methods	Main Findings	Key Limitations	Evidence Role
Sheffield et al., 2016 [39]	Retrospective cohort study	80 NSCLC samples	TMA; PD-L1 IHC using four antibody clones (SP142, E1L3N, 28-8, and RBT-PDL1); *CD274* RNA ISH	RNA–protein relationships varied according to the IHC clone; PD-L1 status was additionally discordant between matched primary tumours and nodal metastases in 22% of cases.	TMA sampling may not fully capture intratumoural heterogeneity; lack of a clinical-response reference standard.	Direct transcript-level evidence
Tsimafeyeu et al., 2020 [40]	Comparative biomarker study	473 NSCLC samples (Stage I–IV); *CD274* RT-PCR available in 437 cases	IHC, RT-qPCR	Depending on the assay and threshold, the probability of IHC negativity among RNA-low/negative cases ranged from 70% to 97%, whereas IHC positivity among RNA-high cases ranged from 9% to 45%.	Biobank-based assay comparison without clinical outcome validation	Direct transcript-level evidence

Authors	Study Design	Cohort	Specimen and Analytical Methods	Main Findings	Key Limitations	Evidence Role
Duncan et al., 2019 [41]	Comparative biomarker study	30 NSCLC cases; 28 successfully evaluable by both assays	IHC, RNA ISH	*CD274* RNA expression was significantly higher in PD-L1 IHC-high than IHC-low NSCLC. RNA ISH showed fair classification accuracy relative to SP263 IHC, but selected a partly different PD-L1-high population.	Small NSCLC cohort; no treatment-response validation.	Direct transcript-level evidence

Authors	Study Design	Cohort	Specimen and Analytical Methods	Main Findings	Key Limitations	Evidence Role
Venina et al., 2022 [42]	Comparative biomarker study	167 non-squamous NSCLC samples	TMA, IHC (22C3, SP263, SP142), RT-qPCR	Low *CD274* RNA expression showed a high negative predictive value for PD-L1 IHC negativity, among RNA-low cases, 98% (51/52) were negative by 22C3 and 92% (48/52) by SP263 at the 1% tumour-cell threshold. Conversely, approximately half of cases with moderate-to-high RNA expression showed <1% tumour-cell staining by 22C3 or SP263.	No clinical outcome validation;	Direct transcript-level evidence

Authors	Study Design	Cohort	Specimen and Analytical Methods	Main Findings	Key Limitations	Evidence Role
Søes et al. 2013 [43]	Retrospective methodological study	10 primary lung adenocarcinomas with 10 matched brain metastases, 10 lymph-node metastases from separate NSCLC cases	RT-qPCR	Commonly used reference genes, including *ACTB*, *GAPDH*, and 18S rRNA, were insufficiently stable in primary and metastatic NSCLC tissue. The most stable combinations were *ACTR3* and *PUM1* or *TBP* and *PUM1* in lung tissue, and *ACTR3* and *SF3A1* or *PUM1* and *IPO8* in lymph node metastases.	Small sample size; lymph-node metastases were derived from different primary tumours; *CD274* and *PDCD1* were not directly assessed.	Methodological evidence

Authors	Study Design	Cohort	Specimen and Analytical Methods	Main Findings	Key Limitations	Evidence Role
Jacobson et al., 2011 [44]	Methodological comparison study	One paired NSCLC specimen: long-term preserved FFPE tissue (>20 years) and matched fresh-frozen tissue from the same patient	RT-qPCR, microarray analysis	Long-term preserved FFPE tissue (>20 years) provided interpretable gene-expression profiles with good agreement with matched fresh-frozen tissue, although overall analytical sensitivity was lower in FFPE material.	Single-patient paired comparison; results are platform- and processing-dependent; FFPE material showed reduced analytical sensitivity; *CD274* and *PDCD1* were not directly assessed.	Methodological evidence

Abbreviations: AD, adenocarcinoma; EBUS-TBNA, endobronchial ultrasound-guided transbronchial needle aspiration; FFPE, formalin-fixed paraffin-embedded; ICI, immune checkpoint inhibitor; ICS, immune checkpoint score; IHC, immunohistochemistry; mIF, multiplex immunofluorescence; MS, mass spectrometry; NPV, negative predictive value; NK, natural killer; NSCLC, non-small cell lung carcinoma; OS, overall survival; PFS, progression-free survival; PPV, positive predictive value; QIF, quantitative immunofluorescence; qPCR, quantitative polymerase chain reaction; RFS, relapse-free survival; RNA-seq, RNA sequencing; RNA ISH, RNA in situ hybridisation; RT-qPCR, reverse transcription quantitative polymerase chain reaction; SCC, squamous cell carcinoma; SCLC, small cell lung carcinoma; TCGA, The Cancer Genome Atlas; TIL, tumour infiltrating lymphocytes; TMA, tissue microarray; TPS, tumour proportion score; WES, whole-exome sequencing.
